# Effect of Preoperative Gum Chewing on Postoperative Nausea and Vomiting in Women Undergoing Robotic Laparoscopic Surgery for Uterine Myomas: A Randomized Controlled Trial

**DOI:** 10.3390/life14121693

**Published:** 2024-12-20

**Authors:** Min Suk Chae, Hyun Jung Koh

**Affiliations:** Department of Anesthesiology and Pain Medicine, Seoul St. Mary’s Hospital, College of Medicine, The Catholic University of Korea, 222, Banpo-daero, Seocho-gu, Seoul 06591, Republic of Korea; shscms@catholic.ac.kr

**Keywords:** preoperative gum chewing, postoperative nausea and vomiting, robot-assisted laparoscopic surgery, women’s health

## Abstract

Background: Postoperative nausea and vomiting (PONV) remains a frequent and uncomfortable complication in women undergoing robotic gynecological procedures. Despite the use of various preventive strategies, PONV continues to negatively impact recovery and increase healthcare expenses. This study aimed to evaluate whether the preoperative use of sugar-free chewing gum could effectively minimize the dependence on anti-emetic drugs in women undergoing robot-assisted laparoscopic surgery for uterine myomas. Methods: In this randomized, single-blind study, 92 adult women scheduled for robot-assisted laparoscopic surgery were enrolled. The participants were randomly assigned to one of two groups: a gum-chewing group, which was instructed to chew sugar-free gum for 15 min in the preoperative holding area, or a control group that did not chew gum. The primary outcome was the requirement for anti-emetic medication within the first hour after surgery, when the patient was in the post-anesthesia care unit (PACU). Secondary outcomes included the overall frequency of anti-emetic use. To assess the intervention’s effectiveness independent of any other factors, no prophylactic anti-emetics were administered during surgery. Results: Of the 92 participants, 89 completed the study, with 44 assigned to the gum-chewing group and 45 to the control group. The gum-chewing group showed a significantly lower rate of PONV, requiring anti-emetic treatment (79.5%), compared to the control group (95.6%). Additionally, the control group demonstrated a higher overall need for anti-emetic medications. Notably, there were no reported adverse effects, such as jaw discomfort, dental injuries, or gastric regurgitation, in either group. Conclusions: Chewing sugar-free gum for 15 min prior to surgery was found to be a safe, simple, and effective method to reduce the occurrence of PONV in women undergoing robot-assisted laparoscopic surgery for benign uterine tumors. This non-invasive intervention reduced the reliance on anti-emetic drugs and improved patient comfort, without introducing risks. These findings suggest that preoperative gum chewing could be routinely implemented in clinical settings to enhance surgical outcomes.

## 1. Introduction

Postoperative nausea and vomiting (PONV) remains a significant concern despite the use of various preventive measures. Among women undergoing laparoscopic gynecological surgeries, the incidence of PONV within the first 24–48 h post-surgery ranges from 50% to 70%, even with the administration of anti-emetics [1,2]. Key risk factors for PONV include the female sex, a history of PONV or motion sickness, non-smoking status, and opioid use after surgery. The presence of multiple risk factors further increases the likelihood of PONV, with the cumulative risk reaching up to 80% when all four factors are present [1]. Beyond causing discomfort, PONV is associated with prolonged stays in the post-anesthesia care unit (PACU) and complications, such as dehydration, electrolyte imbalances, aspiration pneumonia, wound dehiscence, delayed recovery, extended hospital stays, and higher healthcare costs [3,4].

Although robot-assisted laparoscopic surgery is a minimally invasive technique, with benefits such as reduced blood loss, minimal tissue damage, decreased postoperative pain, and shorter hospital stays, PONV remains a significant concern for female patients [5,6]. Factors contributing to this include the use of steep surgical positions, such as the Trendelenburg position, and prolonged intraperitoneal carbon dioxide (CO_2_) insufflation, both of which increase intra-abdominal and diaphragm pressure. Additionally, CO_2_ insufflation can cause peritoneal stretching and irritation, further elevating the risk of PONV [7,8]. While robot-assisted laparoscopic surgery reduces the overall trauma and visceral irritation compared to conventional laparoscopy due to the precision of robotic arms, the surgical characteristics and the female sex, both significant risk factors for PONV, necessitate proactive preventive measures by anesthesiologists, even in patients with a minimal amount of other risk factors.

Chewing gum is often referred to as “false feeding” because the act of chewing occurs without actual food intake. Asao et al. were pioneers in suggesting that gum chewing could serve as an economical and painless non-pharmacological intervention to stimulate intestinal activity [9]. Chewing gum encourages saliva production, helps eliminate oral bacteria and viruses, and enhances gastrointestinal peristalsis, thereby aiding recovery after surgery [10,11]. Recent meta-analyses have indicated that gum chewing following digestive or gynecological surgery can improve the recovery of gastrointestinal function [12,13]. Postoperative gum chewing has been shown to reduce nausea, vomiting, and abdominal distension; accelerate the return of the bowel function, including the first passage of gas, defecation, and bowel sounds; lower the incidence of complications, such as intestinal obstruction; and shorten hospital stays [14]. However, most research has focused on the benefits of gum chewing after surgery. More recent studies have begun to examine the effects of preoperative gum chewing on factors such as patient discomfort (including thirst and anxiety before surgery), postoperative complications like PONV and gastrointestinal dysfunction, and the overall hospital stay [15].

This study investigated the potential of preoperative gum chewing to lower the occurrence of PONV in the immediate postoperative phase among women undergoing robot-assisted procedures for benign uterine tumors, including myomas.

## 2. Patients and Methods

### 2.1. Ethical Considerations

This randomized, single-blind trial received approval from the Institutional Review Board and Ethics Committee at Seoul St. Mary’s Hospital, part of the Catholic University of Korea, on 1 March 2024 (approval number: KC24EISI0138). The study was officially registered with ClinicalTrials.gov (identifier: NCT06446583) on 5 June 2024. Informed consent was obtained, in writing, from all the participants, one day before their scheduled surgery, following the ethical principles outlined in the Declaration of Helsinki. The trial was conducted in compliance with the Consolidated Standards of Reporting Trials (CONSORT) guidelines, ensuring adherence to rigorous ethical and methodological standards throughout the study’s design and execution.

### 2.2. Study Population

Adult female patients scheduled for robot-assisted laparoscopic surgery to address multiple myomas were enrolled in this study. The inclusion criteria required participants to be women, aged 19 to 69 years, undergoing elective robot-assisted laparoscopic surgery for multiple myomas, with an American Society of Anesthesiologists (ASA) physical status of I or II. Patients were excluded if their surgical procedure was converted to a different type, or if they had a history of dental issues (e.g., damage, dentures, loose or crowned teeth), temporomandibular joint (TMJ) disorders or surgeries, prior head-and-neck surgery, emergency surgery, or if they declined to participate. Of the 92 women initially recruited, three were excluded, one due to dental damage and two due to TMJ-related histories. The remaining 89 participants were randomly assigned to two groups: 45 in the non-gum-chewing group and 44 in the gum-chewing group.

### 2.3. Randomization and Blinding

**Figure 1:**
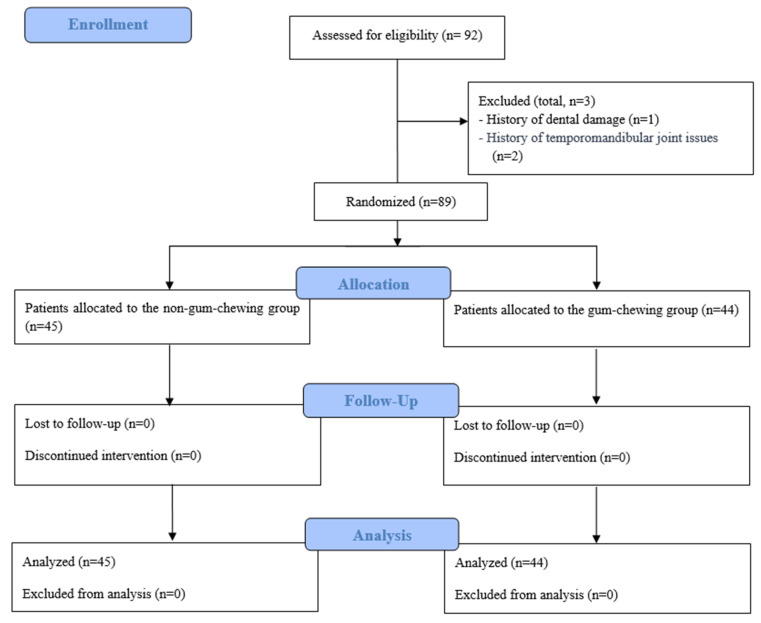
Study flowchart.

The participants were assigned to either the gum-chewing or non-gum-chewing group through stratified block randomization, utilizing a web-based randomization tool (www.random.org accessed on 5 June 2024). To preserve allocation concealment, the research team employed sequentially numbered opaque envelopes to determine the group assignments. These assignments were finalized in the preoperative holding area and discreetly relayed to the sugar-free gum preparation team via sealed envelopes. An anesthesia nurse, uninvolved in the outcome evaluation, prepared the sugar-free gum for the study. To ensure unbiased results, all anesthesiologists and healthcare personnel assessing the postoperative outcomes remained blinded to the participants’ group assignments (Figure 1).

### 2.4. Preoperative Gum-Chewing Protocol

Participants in the gum-chewing group were directed to chew sugar-free mint gum for 15 min while waiting in the preoperative holding area, before being moved to the operating room. The chewing process was closely monitored to ensure proper adherence to the protocol, with participants instructed to chew at a comfortable and steady pace to maximize the intervention’s effect on stimulating gastrointestinal motility. Once the chewing session was complete, the gum was collected by the anesthesia nurse to verify compliance and properly dispose of the gum.

The choice of sugar-free mint gum was intentional, as it is commonly associated with promoting salivary flow and reducing nausea, potentially contributing to the prevention of PONV. While participants were naturally aware of their involvement in the gum-chewing group, every effort was made to ensure that the postoperative care team, including PACU staff, remained blinded to the group allocations. This included concealing the intervention details and ensuring that the gum-chewing activity occurred only in the preoperative area, away from other staff members. These steps were taken to focus on the effectiveness of the gum-chewing intervention in reducing PONV without any external influences, preserving the study’s integrity and reliability.

### 2.5. Outcome Measures

The primary outcome of the study was the need for anti-emetic medications to manage nausea and vomiting during the first postoperative hour in the PACU. As a secondary outcome, the total number of anti-emetic requests made by patients was recorded. To ensure uniformity in assessing the intervention’s effectiveness, no prophylactic anti-emetic drugs were administered during the surgical procedure. In the PACU, when anti-emetic treatment was required, nurses administered a standard initial dose of 0.075 mg of palonosetron hydrochloride (Aloxi; CJ Healthcare, Seoul, Republic of Korea). If the initial dose was insufficient, it was followed by a 4 mg dose of ondansetron (Zofran; Hana Pharm, Seoul, Republic of Korea). This sequential approach ensured consistency in the management of postoperative nausea and vomiting across all the participants.

### 2.6. Robot-Assisted Laparoscopic Surgical Procedures and Anesthetic Management

The da Vinci (Intuitive Surgical Inc., Sunnyvale, CA, USA) robot-assisted laparoscopic surgeries performed in this study included uterine myomectomy (LUM) and total hysterectomy with bilateral salpingo-oophorectomy (TLH with BS), based on discussions between patients and their surgeons. For a myomectomy, patients under general anesthesia were initially positioned in the lithotomy position, then transitioned to the Trendelenburg position. Pneumoperitoneum was established, and trocars were inserted. Robotic arms facilitated the excision of myomas, hemostasis, and suturing of uterine incisions, with myomas removed through small incisions. For a hysterectomy, also performed under general anesthesia, the uterus, fallopian tubes, and ovaries were detached and removed through either the vagina or a small abdominal incision, followed by robotic closure of the vaginal cuff. The precision and control provided by the da Vinci system optimized the surgical outcomes.

General anesthesia was initiated with propofol (2 mg/kg) and rocuronium (0.8–1 mg/kg) to facilitate tracheal intubation. Maintenance of anesthesia was achieved through a continuous infusion of propofol (50–200 µg/kg/min) and remifentanil (0.02–0.10 mg/kg/min), with dosing adjusted individually based on the anesthesiologists’ evaluations. The Minto model was employed for effect-site concentration targeting, ensuring a bispectral index (BIS) of from 40 to 60, while the systolic blood pressure was maintained within 20% of baseline levels. Standard intraoperative monitoring included electrocardiography, pulse oximetry, non-invasive blood pressure measurement, and BIS monitoring. At the conclusion of surgery, the neuromuscular blockade was reversed using sugammadex (4 mg/kg), followed by the administration of 100% oxygen. To ensure the unbiased validation of the study outcomes, no prophylactic anti-emetic medications were administered during the operation. In the PACU, patients with mild PONV were managed with hydration alone, without the use of anti-emetics. For those experiencing moderate to severe PONV, anti-emetics were administered via a stepwise protocol, allowing for up to two doses as required.

For postoperative pain management, intravenous fentanyl (50 µg) was administered to patients reporting peak visual analog scale (VAS) pain scores above 6. Pain assessments were conducted by PACU anesthesiologists and ward physicians, both of whom were uninvolved in the study. All analgesic administrations were meticulously recorded by nursing staff to ensure accurate documentation.

### 2.7. Clinical Variables

Demographic and clinical information was collected prior to surgery, including the patient’s age, the type of procedure (robot-assisted laparoscopic uterine myomectomy [LUM] or total laparoscopic hysterectomy with bilateral salpingo-oophorectomy [TLH with BS]), body mass index (BMI), ASA physical status classification, history of motion sickness or PONV, smoking habits, and comorbidities, such as diabetes mellitus (DM) and hypertension (HTN). The intraoperative data included the duration of surgery, total remifentanil administered, total fluid volume infused, and estimated blood loss. The postoperative data obtained in the PACU included the requirement for additional fentanyl and peak pain scores measured using the VAS. Any adverse effects related to gum chewing, such as jaw or tooth discomfort, injuries, or gastric regurgitation, were meticulously documented.

### 2.8. Sample Size and Statistical Analysis

The sample size for the study was retrospectively calculated based on a preliminary analysis of 20 patients who had undergone robot-assisted laparoscopic surgery for multiple myomas. In regard to the initial findings, the incidence of PONV requiring anti-emetic treatment in the PACU was 70% (7 out of 10 patients) in the non-gum-chewing group and 40% (4 out of 10 patients) in the gum-chewing group. To achieve 80% statistical power, with a type I error rate of 5%, a minimum of 42 participants per group was required, assuming equal allocation (1:1 ratio). To account for an estimated 10% dropout rate, the recruitment target was set at 92 participants, ensuring an adequate sample size for robust and reliable results.

The Shapiro–Wilk test was used to assess the data distribution for normality. Comparisons between the groups for normally distributed variables were conducted using unpaired *t*-tests, while the Mann–Whitney U test was employed for non-normally distributed data. Categorical variables were analyzed using Pearson’s chi-squared test or Fisher’s exact test, as appropriate. Continuous variables are reported as the mean with the standard deviation, and categorical variables as the frequency with the percentage. A *p*-value of less than 0.05 was considered statistically significant. All statistical analyses were performed using SPSS for Windows, version 24.0 (IBM Corp., Armonk, NY, USA).

## 3. Results

The final study cohort comprised 89 female participants, with a mean age of 46.2 ± 11.0 years and an average BMI of 23.5 ± 3.8 kg/m^2^, all of whom underwent surgery for the treatment of multiple myomas. Among these participants, 36 (40.4%) underwent robot-assisted LUM, while 53 (59.6%) received robot-assisted TLH with BS. The ASA classifications indicated that 47 participants (52.8%) were ASA I and 42 (47.2%) were ASA II. Comorbidities included 12 cases of DM (13.5%) and one case of HTN (1.1%). Additionally, 51 participants (57.3%) reported a history of motion sickness and 10 (11.2%) were identified as current smokers.

**Figure 2:**
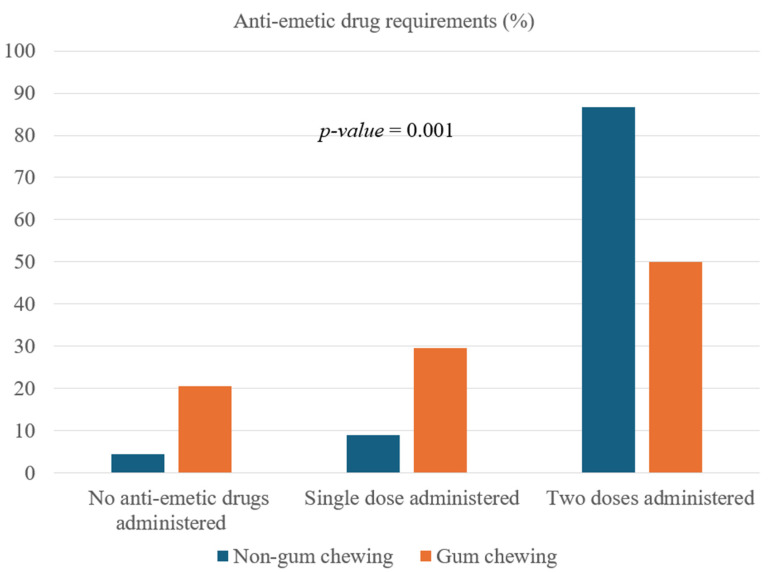
Comparison of anti-emetic drug requirements in the PACU between preoperative gum-chewing and non-gum-chewing groups.

Both the gum-chewing and non-gum-chewing groups showed comparable results across preoperative and intraoperative variables, as well as postoperative pain management metrics, including the administration of rescue fentanyl and peak VAS pain scores (Table 1). However, in the PACU, the non-gum-chewing group exhibited a significantly higher frequency of requiring two doses of anti-emetic medication to manage PONV. In contrast, a greater proportion of patients in the gum-chewing group required either no anti-emetics or only a single dose (Table 2 and Figure 2). Notably, no postoperative complications, such as tooth or jaw discomfort, injuries, or gastric regurgitation, were observed, as confirmed by anesthesiologists who were blinded to the study’s outcomes.

## 4. Discussion

Our study revealed that chewing gum for 15 min before surgery significantly reduces the occurrence of PONV, a condition that often requires anti-emetic intervention, in women undergoing minimally invasive robot-assisted laparoscopic surgeries for benign uterine tumors, such as myomas. This intervention effectively enhanced gastrointestinal motility and mitigated nausea-inducing factors, leading to a notable reduction in the need for anti-emetic medications. Moreover, the gum-chewing approach was well-tolerated, with no reports of adverse events, such as jaw discomfort, dental issues, or gastric regurgitation. Its simplicity, non-invasive nature, and excellent safety profile highlight its potential as a valuable strategy for managing PONV, particularly in clinical settings where minimizing medication use and improving patient comfort are priorities. Additionally, the results demonstrate that even a short chewing duration is sufficient to provide these benefits, offering a practical and easily implementable addition to preoperative care protocols.

Previous systematic reviews and meta-analyses have highlighted the benefits of preoperative gum chewing, indicating that it does not significantly impact the patient’s gastric pH or fluid volume. Studies have shown a reduced incidence of PONV in patients who chewed gum preoperatively compared to those who did not [16]. Chewing gum has been found to alleviate stress, address dry mouth, and stimulate gastrointestinal motility [17,18]. Clinically, preoperative fasting aims to decrease the volume and acidity of the stomach, thereby lowering the risk of gastric regurgitation and pulmonary aspiration during anesthesia [17,19]. Current guidelines generally support a flexible fasting approach, recommending no solid food for 6 h and only clear liquids up to 2 h before elective surgery [20,21]. Since gum chewing is often classified as consuming a solid by anesthesiologists, institutional NPO (nil per os) protocols frequently restrict gum use during fasting, although it is commonly encouraged postoperatively to aid gastrointestinal recovery [4,22,23,24].

The permissibility of gum chewing during preoperative fasting continues to be a subject of debate. Current research indicates that chewing gum does not significantly alter the patient’s gastric volume, with both preoperative and postoperative measurements showing comparable results. A meta-analysis of four studies identified a slight, but statistically significant, increase in the gastric fluid volume linked to preoperative gum chewing. However, this increase is deemed clinically negligible and unlikely to heighten the risk of aspiration. Furthermore, evidence suggests that gum chewing has a minimal effect on gastric acidity, as the patients’ pH levels remain unchanged regardless of the intervention. Importantly, none of the reviewed studies reported instances of gastric regurgitation or aspiration during surgery, reinforcing the safety of gum chewing in this context. These findings suggest that while gum chewing may cause minor changes in the gastric fluid volume, it does not present a meaningful risk during anesthesia or surgery, supporting its potential use as a safe practice during preoperative fasting [25].

Our study demonstrated that a 15 min preoperative gum-chewing intervention significantly reduces the incidence of PONV and the need for multiple doses of antiemetic medications in women undergoing robot-assisted laparoscopic surgery for uterine myomas. Specifically, 50% of the patients in the gum-chewing group required only a single dose of antiemetics or none, compared to 13.3% in the non-gum-chewing group. These findings align with the CHEWY trial [26], which reported that chewing gum was non-inferior to ondansetron in resolving PONV within 2 h postoperatively, achieving comparable effectiveness with fewer side effects and at a lower cost. The mechanism of action, as hypothesized in both studies, includes cephalic-vagal stimulation, which enhances gastrointestinal motility, reduces intra-abdominal pressure, and decreases the accumulation of nausea-inducing metabolites. Furthermore, the safety profile of gum chewing is noteworthy. Neither our study nor the CHEWY trial reported any serious complications, such as aspiration or airway obstruction, supporting the inclusion of gum chewing as a safe adjunct to perioperative care. Importantly, our findings emphasize the practicality of preoperative gum chewing compared to the traditional postoperative approach. The shorter chewing duration of 15 min ensures feasibility in busy preoperative workflows, reducing the likelihood of jaw fatigue or discomfort, while maintaining clinical efficacy. This differs from the duration of 30 min or longer recommended for postoperative interventions. The clinical implications of these findings are significant. Incorporating preoperative gum chewing into multimodal PONV management strategies could enhance patient recovery, particularly in resource-limited settings, where access to expensive antiemetic medications may be constrained. For example, the CHEWY trial noted that the cost of ondansetron could exceed 2% of annual healthcare expenditure in low-income nations, whereas gum chewing offers an alternative at a nearly negligible cost, with broad accessibility. This economic advantage, combined with its effectiveness and safety, positions gum chewing as a valuable component of enhanced recovery protocols for minimally invasive surgeries. Future research should aim to standardize preoperative gum-chewing protocols, including optimal durations and timings relative to surgery. Additionally, comparative studies examining the synergistic effects of preoperative gum chewing and pharmacological antiemetics may provide further insights into maximizing patient outcomes, while minimizing medication use.

Chewing gum prior to surgery may play a significant role in promoting postoperative gastrointestinal recovery [27]. This practice activates both mechanical and chemical receptors in the oral cavity, initiating a cascade of physiological responses. These include the stimulation of digestive enzyme secretion and an increase in gastrointestinal motility, which helps to prepare the digestive system for recovery after surgery. Additionally, chewing gum triggers the release of gastrin and other gastrointestinal hormones, which further support digestive processes. These benefits mirror those observed with gum chewing implemented during the postoperative period, emphasizing its utility at different stages of surgical care [15]. The activation of the vagus nerve during gum chewing enhances acetylcholine release, which is crucial for stimulating gastric peristalsis. This process is accompanied by the dilation of splanchnic blood vessels and improved gastrointestinal blood flow, all of which contribute to alleviating postoperative bloating, constipation, and discomfort associated with delayed bowel function. Together, these effects not only expedite gastrointestinal recovery, but also reduce associated pain and enhance the patient’s overall postoperative well-being [14,18]. Additionally, preoperative gum chewing has demonstrated no detrimental effects on the gastric content volume or acidity, indicating that it does not elevate the risk of aspiration during surgery. This favorable safety profile positions preoperative gum chewing as a practical and effective approach for reducing PONV and decreasing the reliance on anti-emetic medications in women undergoing robot-assisted laparoscopic surgeries for benign uterine tumors, such as myomas. By mitigating PONV through this straightforward, non-invasive method, preoperative gum chewing provides both clinical advantages and patient-centered benefits, facilitating recovery without introducing the risks or complications often associated with pharmacological treatments.

The European Society of Anesthesiology advises that surgical procedures should not be postponed or canceled solely because of preoperative gum chewing, consuming a boiled sweet, or smoking shortly before anesthesia induction [21]. However, it is essential to ensure that gum is removed before anesthesia is administered to mitigate the risk of airway obstruction during the procedure. To further enhance safety, it is recommended that preoperative assessment checklists include a specific question about gum chewing [28]. This would enable healthcare providers to identify and address any potential risks systematically. For patients who have chewed gum during the pre-anesthetic fasting period, it should be discarded before entering the operating room. In this study, a nurse in the preoperative holding area ensured that participants in the gum-chewing group removed their gum before transfer. This precaution helped minimize the risk of complications, such as aspiration or airway obstruction, thereby maintaining safety during anesthesia and surgery. By implementing standardized protocols for managing preoperative gum chewing, these risks can be effectively managed without causing delays or disruptions to surgical schedules.

Among the ongoing discussions on the efficacy of gum chewing for PONV management, a recent study highlighted that gum chewing alone cannot replace anti-emetics when PONV occurs, even when prophylactic anti-emetics are administered preoperatively [29]. However, our findings demonstrate that preoperative gum chewing significantly reduces the need for anti-emetic medications, even in the absence of prophylactic administration. This suggests that preoperative gum chewing is as effective as postoperative gum chewing in managing PONV. Despite their comparable effects, the key distinction lies in the timing: preoperative gum chewing proactively stimulates gastrointestinal motility and reduces nausea triggers before surgery, while postoperative gum chewing primarily aids in restoring the gastrointestinal function disrupted by surgical trauma [16,29,30]. Regarding duration, the existing literature generally highlights that gum chewing for 30 min or longer is necessary to achieve optimal results in regard to PONV management. However, our study found that even a short chewing period of 15 min preoperatively yielded significant benefits. This finding is particularly meaningful as it not only minimizes the time required for PONV prevention, but also aligns with dental recommendations to avoid prolonged chewing, which could lead to fatigue or strain on the jaw muscles and temporomandibular joint. Furthermore, shorter preoperative chewing intervals may improve patient compliance, as they are less burdensome and can be easily integrated into pre-surgical routines [31,32]. Future studies should further explore the comparative benefits of different durations and timings in terms of gum chewing to establish a standardized protocol for clinical practice.

Our study has several limitations that should be acknowledged. The relatively small sample size may have influenced the statistical power of our findings, increasing the potential for both type I (false positive) and type II (false negative) errors. While the results demonstrated a statistically significant reduction in PONV with preoperative gum chewing, a larger sample size would enhance the robustness of these findings and reduce the risk of statistical inaccuracies. Another limitation lies in the homogeneity of the study population. All participants were women undergoing robot-assisted laparoscopic surgery for uterine myomas, which restricts the generalizability of our findings to other patient populations or surgical procedures. Future studies should broaden the inclusion criteria to incorporate more diverse patient demographics and surgical types, ensuring that the intervention’s efficacy can be validated in a wider clinical context. Additionally, this study focused solely on the acute postoperative period within the PACU. Long-term outcomes, such as the impact of gum chewing on extended hospital stays, recovery times, and overall healthcare costs, were not assessed. Investigating these aspects would provide a more comprehensive understanding of the clinical utility of preoperative gum chewing. Lastly, while we adhered to rigorous methodological standards, the single-center design may have introduced an element of institutional bias that could limit the applicability of our findings to other healthcare settings. Despite these limitations, our study provides meaningful preliminary evidence that preoperative gum chewing is a safe, non-invasive, and cost-effective intervention for reducing PONV. These results lay the groundwork for larger, multicenter trials that can confirm the findings, explore broader applications, and assess the long-term benefits of this simple, yet innovative, approach.

## 5. Conclusions

Chewing gum for a brief duration before surgery has been proven to be a safe, non-invasive, and effective method for reducing PONV in women undergoing minimally invasive robot-assisted surgeries for uterine myomas. This straightforward preoperative intervention significantly lowered the incidence of PONV and the reliance on anti-emetic medications, improving patient comfort and optimizing postoperative care. Additionally, the reduction in PONV may translate into substantial cost savings and shorter hospital stays. By shifting the focus to include preoperative gum chewing alongside postoperative strategies, this approach holds potential for broader implementation across diverse surgical specialties, enhancing recovery outcomes and overall patient satisfaction.

## Figures and Tables

**Table 1:** Demographics of the preoperative gum-chewing and non-gum-chewing groups.

Group	Non-Gum Chewing	Gum Chewing	*p*-Value
n	45	44	
***Preoperative findings***			
Age (years)	46 ± 11	47 ± 11	0.737
Operation type			
Robot-assisted TLH with BS	18 (40.0%)	18 (40.9%)	0.93
Robot-assisted LUM	27 (60.0%)	26 (59.1%)	
Body mass index (kg/m^2^)	23.4 ± 3.6	23.7 ± 4.1	0.683
ASA physical class			
Class I	24 (53.3%)	23 (52.3%)	0.92
Class II	21 (46.7%)	21 (47.7%)	
History of motion sickness/PONV	26 (57.8%)	25 (56.8%)	0.927
Current smoker	7 (15.6%)	3 (6.8%)	0.192
Diabetes mellitus	5 (11.1%)	7 (15.9%)	0.508
Hypertension	0 (0.0%)	1 (2.3%)	0.494
***Intraoperative findings***			
Surgery duration (min)	155.6 ± 77.2	149.0 ± 63.7	0.66
Total remifentanil infusion (mg)	0.6 ± 0.4	0.6 ± 0.3	0.753
Total fluid infusion (mL)	992.3 ± 794.3	832.4 ± 545.8	0.273
Total hemorrhage (mL)	216.3 ± 227.5	185.3 ± 219.2	0.515
***Pain findings in the PACU***			
Rescue fentanyl infusion (mcg)	54.4 ± 14.4	52.3 ± 10.5	0.42
Peak VAS	4.8 ± 0.6	4.5 ± 1.0	0.073

**Abbreviations:** TLH with BS, total laparoscopic hysterectomy with bilateral salpingo-oophorectomy; LUM, laparoscopic uterine myomectomy; ASA, American Society of Anesthesiologists; PONV, postoperative nausea and vomiting; VAS, visual analog scale; PACU, post-anesthesia care unit. Values are expressed as mean ± SD and number (proportion).

**Table 2:** Comparison of anti-emetic drug requirements in the PACU between preoperative gum-chewing and non-gum-chewing groups.

Group	Non-Gum Chewing	Gum Chewing	*p*-Value
n	45	44	
Anti-emetic drug requirement			0.001
No anti-emetic drugs administered	2 (4.4%)	9 (20.5%)	
Single dose administered	4 (8.9%)	13 (29.5%)	
Two doses administered	39 (86.7%)	22 (50.0%)	

**Abbreviation:** PONV, postoperative nausea and vomiting; PACU, post-anesthesia care unit. Values are expressed as numbers (proportion).

## Data Availability

The data are contained within the article.

## Abbreviations

PONV: postoperative nausea and vomiting; PACU, post-anesthesia care unit; RALS, robot-assisted laparoscopic surgery; ASA, American Society of Anesthesiologists; TMJ, temporomandibular joint; VAS, visual analog scale; DM, diabetes mellitus; HBP, hypertension; BMI, body mass index; LUM, laparoscopic uterine myomectomy; TLH with BS, total laparoscopic hysterectomy with bilateral salpingo-oophorectomy; NPO, nil per os; CONSORT, Consolidated Standards of Reporting Trials; BIS, bispectral index.
